# Cryptocurrency price drivers: Wavelet coherence analysis revisited

**DOI:** 10.1371/journal.pone.0195200

**Published:** 2018-04-18

**Authors:** Ross C. Phillips, Denise Gorse

**Affiliations:** Department of Computer Science, University College London, London, United kingdom; University of the Basque Country, SPAIN

## Abstract

Cryptocurrencies have experienced recent surges in interest and price. It has been discovered that there are time intervals where cryptocurrency prices and certain online and social media factors appear related. In addition it has been noted that cryptocurrencies are prone to experience intervals of bubble-like price growth. The hypothesis investigated here is that relationships between online factors and price are dependent on market regime. In this paper, wavelet coherence is used to study co-movement between a cryptocurrency price and its related factors, for a number of examples. This is used alongside a well-known test for financial asset bubbles to explore whether relationships change dependent on regime. The primary finding of this work is that medium-term positive correlations between online factors and price strengthen significantly during bubble-like regimes of the price series; this explains why these relationships have previously been seen to appear and disappear over time. A secondary finding is that short-term relationships between the chosen factors and price appear to be caused by particular market events (such as hacks / security breaches), and are not consistent from one time interval to another in the effect of the factor upon the price. In addition, for the first time, wavelet coherence is used to explore the relationships between different cryptocurrencies.

## 1 Introduction

Cryptocurrencies are receiving a new wave of media attention. Although some people are only just hearing about cryptocurrencies, they have existed in their current form for several years—the most well-known, Bitcoin, was introduced in late 2008 [1]. Numerous studies have attempted to provide understanding of how cryptocurrency prices can be predicted, many of these focussing on monitoring online factors, especially those derived from social media activity (given social media’s success in predicting stock prices [2]). For example, relevant (e.g. “Bitcoin”) Google search volumes and Wikipedia views are reported to have a bidirectional positive relationship with the Bitcoin price [3]; a self-reinforcing positive feedback loop between the volume of Twitter messages and the Bitcoin price was observed in [4]; polarization of opinions in Twitter messages was found to be a leading indicator of price in [5]; and usage of selected dedicated online forums was analysed for its ability to predict price fluctuations in [6]. Most recently, social media factors, derived from a relatively unexplored social media platform (Reddit), were used by the current authors as inputs to a hidden Markov model, successfully detecting epidemic-like price bubbles [7].

Although relationships between online factors and price may be present for certain time intervals, it is apparent from our inspection of previous work [8] using wavelet coherence [9] that relationships between particular factors and the Bitcoin price are not consistently present; it is the intention of the current study to revisit and extend the work of [8] (using a longer data period and additional factors), and in addition to use wavelet coherence to investigate relationships between different cryptocurrency price series. Wavelet coherence, which can monitor changing temporal relationships occurring over the short, medium, and long term, has been used in the financial literature to track relationships between stock indices [10], commodities [11], cross-asset behaviour [12] and between social media and stock prices [13]. In addition to the Bitcoin-focussed wavelet coherence work of [8], wavelet analysis has been used to identify co-movement between Bitcoin and, separately, global uncertainty [14] and regional markets [15].

It is our hypothesis that a cryptocurrency’s relationship with potentially relevant online usage factors is dependent on market regime. Market regimes have previously been observed in cryptocurrency markets, particularly bubbles [7, 16], but also bull and bear markets [17]. To validate our above hypothesis this work combines wavelet coherence with the application of a test for bubbles, this combination of methods being used to determine whether relationships strengthen during bubble regimes. This is done here not only for Bitcoin but for other cryptocurrencies; this is the first time a wavelet based factor analysis has been carried out for cryptocurrencies other than Bitcoin, with results which may be of interest to those intending to construct a cryptocurrency portfolio. The additional work done here investigating possible relationships between cryptocurrencies may be of particular importance to those looking to diversify risk.

## 2 Materials and methods

### 2.1 Data

This section details the data used in this work; all data collection was undertaken while following the appropriate terms of service and privacy conditions of each respective data source outlined below.

#### 2.1.1 Cryptocurrency price data

Like other recent work [6, 7], this work will consider a cryptocurrency universe beyond Bitcoin. Four cryptocurrencies will be examined: Bitcoin, Ethereum, Litecoin, and Monero. When examining other financial markets (e.g. equities and commodities), work is required to pre-process data to avoid spurious correlations caused by exchange holidays and other intervals when trading is not possible [10]. Cryptocurrency markets are unusual in the sense that they operate 24 hours a day, 7 days a week, with no planned closures, and as such, this is not an issue. However, although cryptocurrency exchanges do not have planned closures, they are prone to unscheduled outages where trading is not possible on a particular exchange. Furthermore, historically cryptocurrency trading exchanges have been notoriously bad at remaining operational with, at one point, 45% of cryptocurrency trading exchanges having shut down [18]. For these reasons aggregated trading-related data from a number of exchanges is used, where possible. Table 1 outlines the data source and time interval considered for each cryptocurrency. The time interval for each cryptocurrency is chosen so as to contain all its major price movements.

**Table 1:** Time intervals considered for each cryptocurrency.

Cryptocurrency	Source	Start Date	End Date
Bitcoin	BraveNewCoin aggregated index	2010-09-10	2017-05-31
Ethereum	BraveNewCoin aggregated index	2015-08-08	2017-05-31
Monero	BraveNewCoin aggregated index	2014-05-19	2017-05-31
Litecoin	BTC-E price	2012-07-13	2017-05-31

The BraveNewCoin aggregated index is chosen as the source of data for Bitcoin, Ethereum and Monero. The BraveNewCoin aggregated index is not used for Litecoin as their index for Litecoin only starts in April 2014 and misses earlier price action. Instead, Litecoin data is retrieved from the BTC-E time series. It should be noted BTC-E has recently (25th July 2017) been shut down by US authorities, however this is after the data interval examined. It has been observed that price differences do exist between cryptocurrency exchanges [19], and it is expected the BTC-E price over time will be different to other exchanges, however with the possibility of exchange arbitrage, prices on different exchanges are reasonably similar.

**Fig 1:**
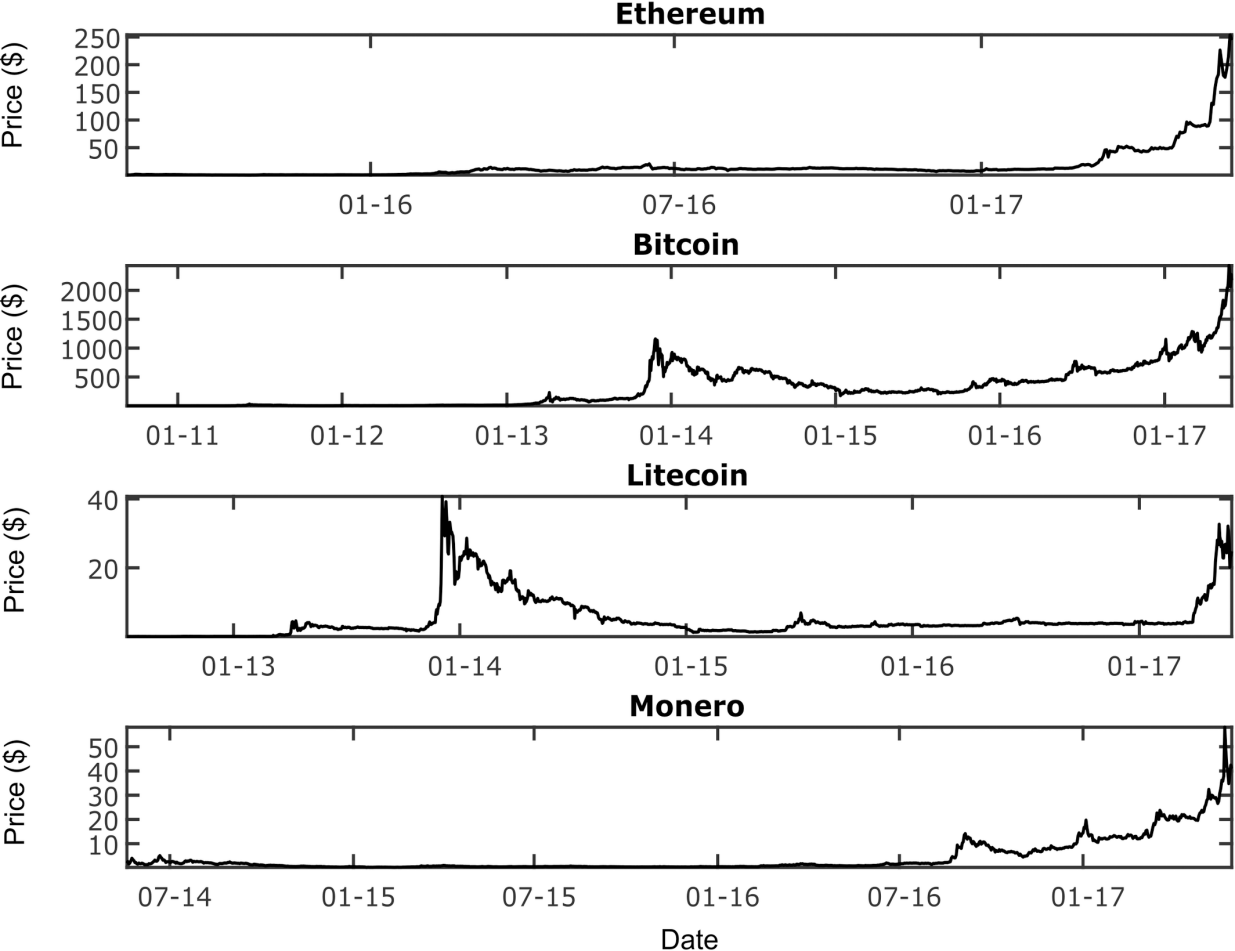
Price series for each cryptocurrency considered (each cryptocurrency priced in USD).

Raw time series can be multi-modal. This is especially apparent for financial asset price time series, as prices are likely to locate around psychological supports and resistances [20]. Multi-modal distributions are not ideal for use in wavelet analysis, and it is advised to transform the time series to avoid such distributions [21]. As commonly done elsewhere [10, 11], log returns are used instead of the raw time series, resulting in unimodal distributions nearer the normal distribution. The same transformation is applied to all online metric time series, to the same affect. As a result all the time series under examination can be considered as growth rates rather than absolute amounts; an important design decision as one would expect peaks in growth rates to lead peaks in absolute values (and as such could be interpreted wrongly as a leading relationship, if one time series was growth rates and another absolute values). Fig 1 shows the price series evolution for each cryptocurrency considered.

#### 2.1.2 Social media factors derived from Reddit

Reddit, an online social media platform, is a collection of communities relating to different topics. Unlike other social networks where the focus is on interacting with those with which one already has a shared connection, people on Reddit congregate together based on their shared interest in a particular topic. To use its own terminology, Reddit is separated into different *subreddits*, where a subreddit is an area of Reddit dedicated to a particular topic. With an account, it is possible to subscribe to as many subreddits as desired, and post and comment in those subreddits. Each major cryptocurrency has its own subreddit. Subreddits are commonly used by the development teams of a particular cryptocurrency to engage with the community, debate technical issues, and distribute news. There have also been cases where important time sensitive news (hacks/code bugs) has first appeared as a Reddit post by a community member, before being discussed publically by the development team. All of the cryptocurrencies considered have multiple associated subreddits; however in each case this work will consider only the largest discussion group (respectively, /r/bitcoin, /r/ethereum, /r/monero, /r/litecoin).

Various factors can be used to monitor usage of a subreddit. Due to the promising trading strategy generated from the factors chosen in [7], these same factors will be examined here. *Posts per day* indicates the number of posts made on a particular subreddit, per day. (this factor does not include comments made in response to particular posts). *Subscriber growth* indicates the number of new subscribers that a subreddit receives, per day. *New authors* indicates the number of new authors posting on a particular subreddit, per day. This current work will aim to confirm the relationship the factors identified have with price in a model-free environment, rather than with the use of a trading strategy which can potentially introduce ambiguity relating to individual factor contributions.

Posts per day and new authors can be retrieved from each subreddit programmatically; each post is timestamped, so historical time series can be generated by iterating through the posts. Posts per day are used here rather than comments per day. Each post on Reddit can have a number of associated comments in a one-to-many type relationship. However, examples exist where huge numbers of comments are generated that are unrelated to market activity; for example, sometimes people give away small amounts of cryptocurrency to everyone who comments with their public blockchain wallet address; this causes a huge spike in comments (wavelet coherence between comments per day and price were also generated, but as was expected showed less significant relationships than posts per day and price).

**Fig 2:**
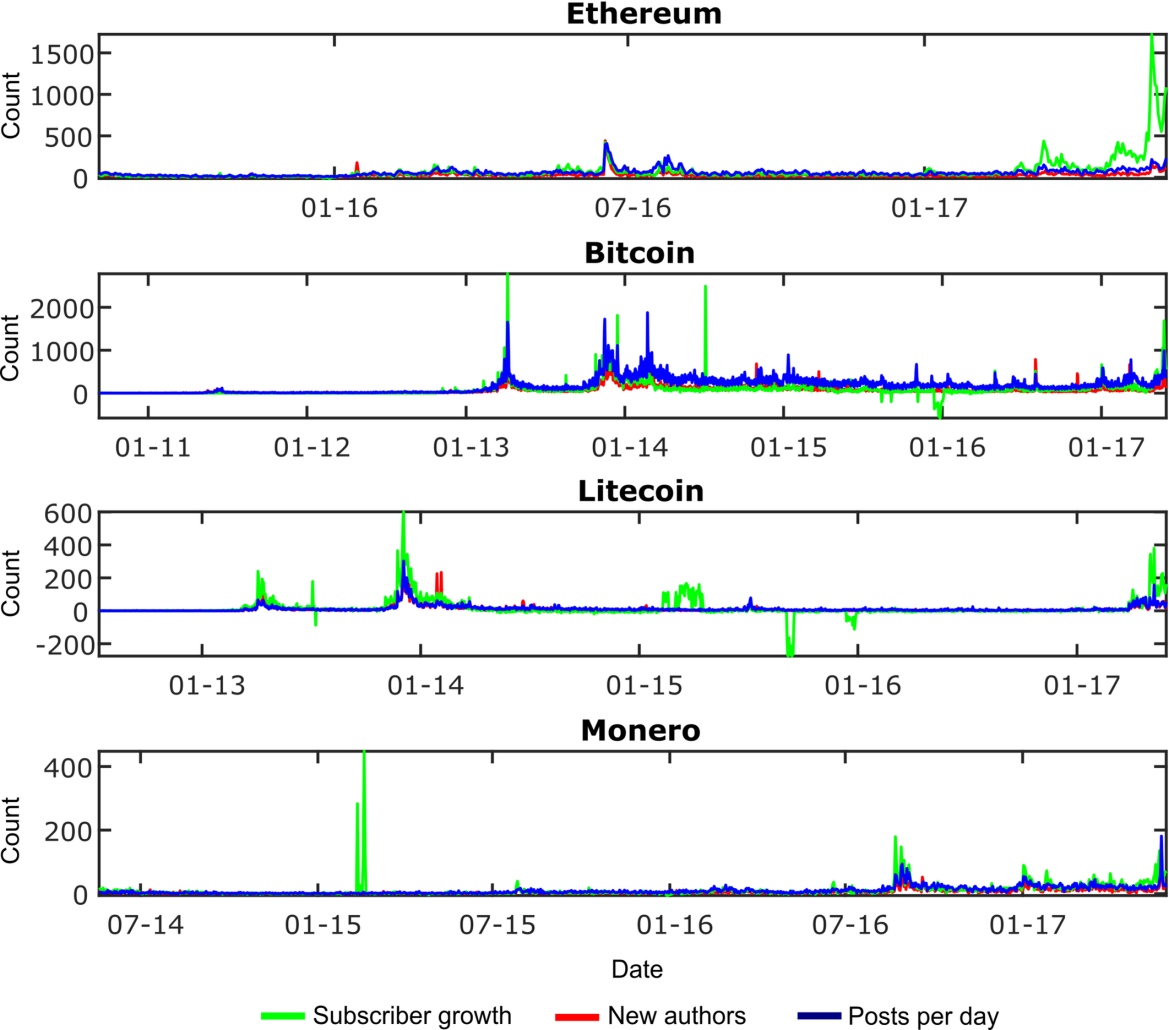
Social media metrics for each cryptocurrency considered.

Subscriber growth is harder to track than the other metrics. Only the current subscriber count is displayed for a particular subreddit, and historical data cannot be rebuilt retrospectively as subscribers do not have a visible historical imprint. A third-party website, RedditMetrics (http://redditmetrics.com/), has been retrieving and storing real-time subscriber counts; however their data on the particular subreddits of interest only reaches back to 2012 so the subscriber growth analysis can only start at this point. Fig 2 shows the three social media metric time series for each cryptocurrency; note that subscriber growth is the only metric that can have negative associated values, caused by more users unsubscribing than subscribing on a particular day.

#### 2.1.3 Google search volume

The volume of searches for particular terms is retrieved from the Google Trends service, a service provided by Google to give an insight into the popularity of different search terms over time. Search volumes returned from Google Trends are scaled from 0 to 100, where 100 represents the highest search volume within the time frame queried. In this work, the search term considered is the name of each cryptocurrency; for example, the volume of searches for “Bitcoin”, “Ethereum” etc.

Google Trends returns data with different granularity depending on the historical time interval queried: daily search volumes are returned for queries under 90 days and weekly search volumes for queries of length over 90 days. However it is possible to reconstruct daily data for long time intervals using a combination of daily and weekly data and the method described and validated by [4]: Daily data is retrieved in buckets of under 90 days, and weekly data is also retrieved for the complete time interval of interest. Then using the daily data, the percentage change of each day in a week from the first day of the week is calculated; these percentage changes are then applied to the weekly data to build a daily time series over a longer period.

#### 2.1.4 Wikipedia

Each major cryptocurrency has its own Wikipedia page providing an introduction to the cryptocurrency. Monitoring Wikipedia views has been seen to be a good way to track the number of new users learning about a cryptocurrency [22], and may offer different insights to the other online factors considered, being focussed primarily on less knowledgeable users.

There is not one single location for Wikipedia views data over the historical data interval required. Wikipedia views data from the start of 2015 onwards can be retrieved using the official *mwviews* python library which connects to Wikipedia’s pageview API. Previous historical data can be retrieved in one month buckets from a separate website (http://stats.grok.de). Data was programmatically retrieved here from both sources, and then merged to produce a single time series.

### 2.2 Methodology

#### 2.2.1 Wavelets

A comprehensive explanation of wavelet methodologies can be found for example in [10, 11, 21]; this section aims to provide an overview based on the presentation in these papers.

Wavelets are wavelike functions used to transform signals into a representation which has time and frequency domain components. Visually wavelets appear as wave-like oscillations with an amplitude that starts at zero, increases, then returns to zero. Another way to consider a wavelet is as a bandpass filter, which can be applied to a time series under investigation, letting through only components of the time series within a finite range of frequencies to different extents depending on the energy spectrum of the wavelet. Wavelets take the form:

$$\psi_{u,s}(t)=\frac{1}{\sqrt{s}}\psi \left(\frac{t-u}{s}\right)$$

The *u* parameter specifies the location of the wavelet. The scale parameter *s* refers to the width of the wavelet, indicating how stretched or dilated the wavelet is while retaining the same wavelike shape. Larger values of *s* increase the width of the wavelet, and therefore more of the observed time series is considered, but granularity of the observation is reduced meaning a higher-level view of the time series is taken. Low scales will allow for analysis of (higher frequency) short-term dynamics of the time series under consideration, whereas high scales will allow for analysis of (lower frequency) long-term dynamics. If the wavelet and time series follow a similar pattern at a specific temporal location and scale, then a large transform value is generated. If the wavelet function is applied in a continuous fashion, as done in this work, this is referred to as *continuous wavelet transform*. The continuous wavelet transform is defined as

$$W_{x}(u,s)=\int_{-\infty }^{+\infty }x(t)\frac{1}{\sqrt{s}}\psi^{*}\;\left(\frac{t-u}{s}\right)\;dt$$

where ψ* is the complex conjugate of *ψ*. There are many examples of functions that can be categorised as a wavelet. As has been used in similar previous financial applications [10, 11], the Morlet wavelet has been used here. It is made up of a normalisation factor, complex sinusoid, and Gaussian bell curve. It is essentially a sine wave multiplied point by point by a Gaussian. The Morlet wavelet is defined as

$$\psi^{Μ}(t)=\frac{1}{\pi^{1/4}}e^{i\omega_{0}t}e^{-t^{2}/2}$$

where *ω*_0_ is chosen to be 6, a good choice for feature extraction purposes [21] and is a commonly chosen value in similar pursuits [10, 11]. Continuous wavelet transforms are useful when considering a time series and breaking down and examining its constituent waveforms. It is also possible to use another wavelet transform, the *cross wavelet transform*, to examine two time series with the aim of identifying locations where similar correlations with a particular wavelet exist. This is defined for two continuous wavelet transforms, *W*_*x*_(*u*, *s*) and *W*_*y*_(*u*, *s*), as

$$W_{x,y}(u,s)=W_{x}(u,s)\;{W^{*}}_{y}(u,s)$$

where * denotes the complex conjugate. Regions that have high values in both continuous wavelet transform will result in high cross wavelet power (|*W*_*x*,*y*_(*u*, *s*)|).

As in previous work [8, 10, 11], it is of more interest whether the time series being considered co-move, than whether they produce large cross wavelet transform values, and hence *wavelet coherence* is utilised for this purpose. Wavelet coherence is defined as

$$R^{2}(u,s)=\frac{{\left|S(s^{-1}W_{x,y}(u,s))\right|}^{2}}{S(s^{-1}{\left|W_{x}(u,s)\right|}^{2})S(s^{-1}{\left|W_{y}(u,s)\right|}^{2})}$$

where S is a smoothing operator applied in both the time and frequency domain (the smoothing operator used in this work is described by [21]). Wavelet coherence is the ratio of the cross wavelet power to the product of the individual wavelet power, comparable to the squared coefficient of correlation; essentially this is providing the correlation coefficient around each moment in time and for each frequency. It can be used to identify regions in time-frequency space where the two time series being examined move in a similar way, though they do not necessarily display high power. A map of phase differences between the signals can also be obtained. This can be used to identify the lag between the two time series (which series is leading and which series is lagging).

#### 2.2.2 Further details and interpretation of wavelet coherence scalograms

**Fig 3:**
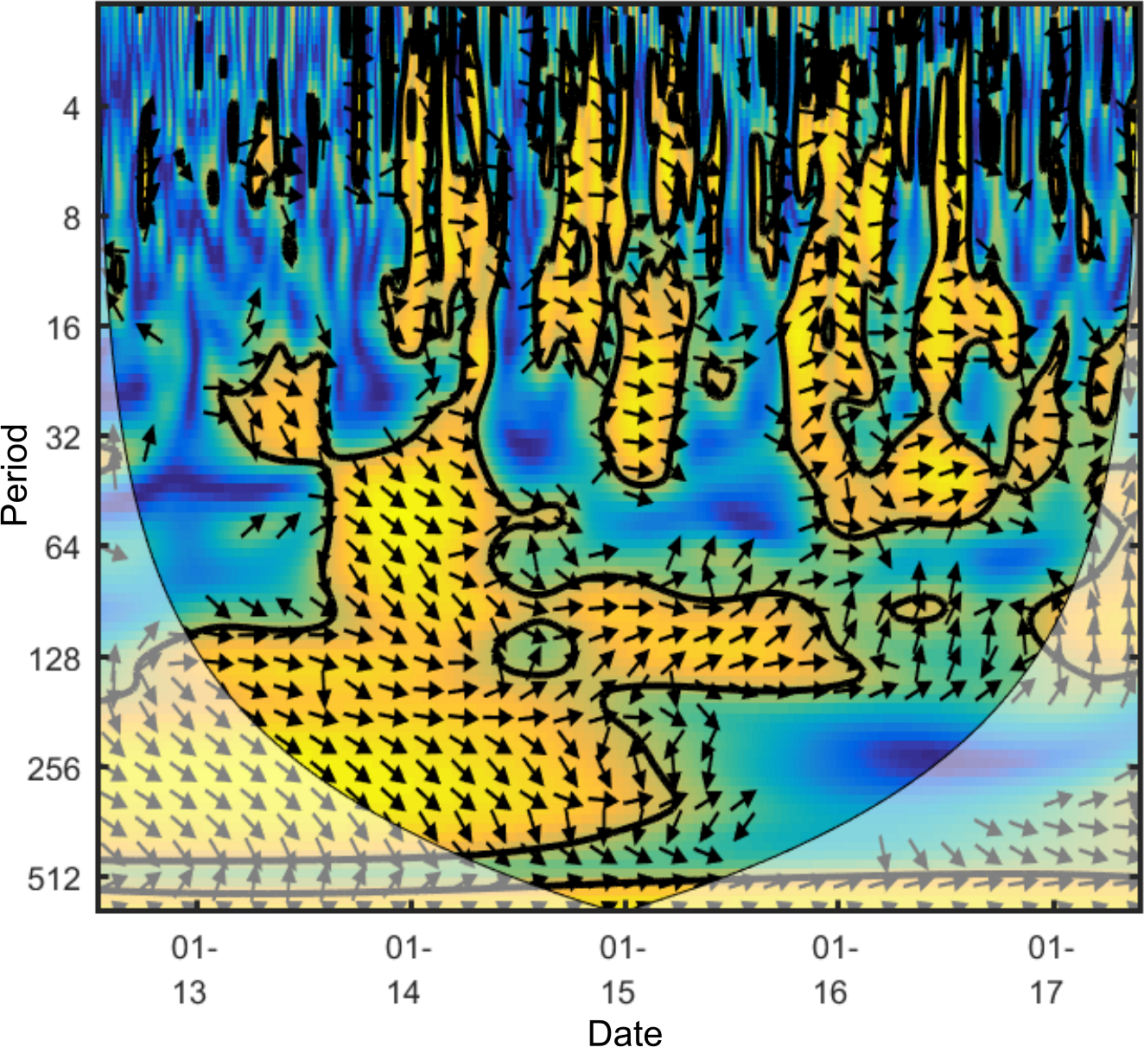
Example wavelet coherence scalogram.

Fig 3 shows an example wavelet coherence scalogram (the wavelet coherence scalogram for Bitcoin and Litecoin which will be analysed later). All following scalograms use the cross wavelet and wavelet coherence software provided by A. Grinsted [21].

The horizontal axis shows the time; relationships positioned towards the leftmost area of a diagram occurred at the start of the data interval considered, and those at the rightmost end occurred at the end of the data interval considered. The vertical axis shows the period; lower period bands (higher frequencies) are shown near the top and higher bands (lower frequencies) are near the bottom. Lower bands would be of interest to investors with short term horizons, whereas higher bands would be of interest to investors with longer term horizons.

Wavelet coherence plots as above highlight areas in the time-frequency space where the two series co-move. The warmer the colour, the higher the coherence (which can be interpreted as correlation) at that location in the time-frequency space; the colours used in this work range from dark blue (0, no coherence) to yellow (1, strong coherence). Statistically significant areas of coherence are surrounded by a thick black line.

The direction of the *oriented arrows* displays two things: the correlation, and which time series is leading the relationship at that point. An arrow pointing left is *anti-phase*, meaning the two time series are negatively correlated at this location. An arrow pointing right is *in-phase* meaning the two time series are positively correlated at this location. A downward arrow means the first time series is leading the second whereas an upwards arrow means the second time series is leading the first. In Fig 3 it is possible for example to see arrows pointing southeast around time in 2013/2014 and period band of 256–512 days; this can be interpreted as the two time series being positively correlated at that time, with the first series (Bitcoin price) leading the second series (Litecoin price). In the later scalograms that include an online factor and price, the online factor will always be the first time series and the price series the second, meaning a downward arrow will indicate that the factor is leading the price.

At each point information from neighbouring data is used. As the time series considered are finite, the areas at the start and end of the data (especially at higher period bands) will not have all the data required. One solution to make computation possible, chosen here, is to pad the time series with zeros where required. However, the zero padding will impact the reliability of the results. It is standard to use a *cone of influence* to represent this difference in reliability of results. Pale colours represent those areas outside the cone of influence with less reliable results (as seen on Fig 3). Higher period bands require more data for computation resulting in the cone shape.

#### 2.2.3 Bubble detection using the GSADF test

In order to provide a methodology to detect bubbles in time series, Phillips, Wu, and Yu [23] proposed the *supremum augmented Dickey-Fuller* (SADF) test. This applies a series of right-tailed unit root tests to expanding windows of a time series (with a fixed start date), defined by

$$SADF(r_{0})=\underset{r_{2}\in [r_{0},1]}{\sup}{ADF}_{0}^{r_{2}}$$

where r_2_ is the final data point to be considered in each window, starting at r_0_ which is a fraction representing the smallest allowed window size and expanding to 1(the complete data set).

The SADF test finds the largest ADF statistic from all the windows considered. If this value exceeds a critical value, the null hypothesis can be rejected, and it is deemed the series displays explosive behaviour in at least one of the windows (taken as indication of a bubble occurring).

Although this test successfully detects single isolated bubbles, Phillips, Shi, and Yu [24] acknowledge it may suffer from reduced discriminatory power when applied to time series with multiple occurrences of bubbles. To overcome this weakness, a further enhancement was proposed, as a new method, called a *generalized supremum ADF* (GSADF) test. This test allows both the start and end points of data subsets to vary, which in turn enables the identification of multiple bubble regimes in one observed time series. The GSADF test is defined by

$$GSADF(r_{0})=\underset{\begin{array}{c} r_{2}\in [r_{0},1],\\ r_{1}\in [0,r_{2}-r_{0}] \end{array}}{\sup}{ADF}_{r_{1}}^{r_{2}}$$

Whereas in the original SADF test the starting value of the window, r_1_, was fixed to 0, in the GSADF test the starting point can now vary from 0 to r_2_ − r_0_ (this is the last possible starting point, near the end of the data set, that allows the test to be run on the minimum window size).

Further to the above, better results were found [24] compared to SADF when using a backward expanding window, which they introduced as *backward SADF* (BSADF). This performs the same supremum ADF test, but this time with a fixed ending point, r_2_, and backwards expanding window:

$${BSADF}_{r_{2}}(r_{0})=\underset{r_{1}\in [0,r_{2}-r_{0}]}{\sup}{ADF}_{r_{1}}^{r_{2}}$$

Combining the BSADF with the GSADF test allows the r_2_ value to vary while still using a backward expanding window. r_2_ starts at the smallest possible window size, and moves one point at a time towards the end of the time series.

$$GSADF(r_{0})=\underset{\begin{array}{c} r_{2}\in [r_{0},1] \end{array}}{\sup}{BSADF}_{r_{2}}(r_{0})$$

The GSADF method can be used to date stamp the start and end of bubble regimes. At each point of r_2_, the BSADF statistic is generated. The start of a bubble is defined as the first r_2_ value that generates a BSADF value larger than the appropriate critical value (the null hypothesis of a unit root in the time series is rejected in favour of a mildly explosive alternative). The end of the bubble is the first r_2_ after the start point such that the BSADF statistic is smaller than the critical value. Finite sample critical values are obtained via Monte Carlo simulation of a Wiener process, approximated by the partial sums of N(0,1). Generation of these values for the current work proved to be computationally expensive. To achieve this in a reasonable time, a cloud-based infrastructure was used, enabling the work to be parallelised and provided a speed up of around 46 times compared to calculating the values on a single CPU. Convenient integration between Matlab and Google Cloud was achieved through using a software called Techila Technologies.

As noted elsewhere by a prominent author in the area [25], there is not a widely accepted or consistent definition of the term “bubble”. The GDASF test used here assumes a bubble is any time series interval which deviates from a random walk to become explosive.

## 3 Results and discussion

### 3.1 Coherence between cryptocurrencies and online factors

**Fig 4:**
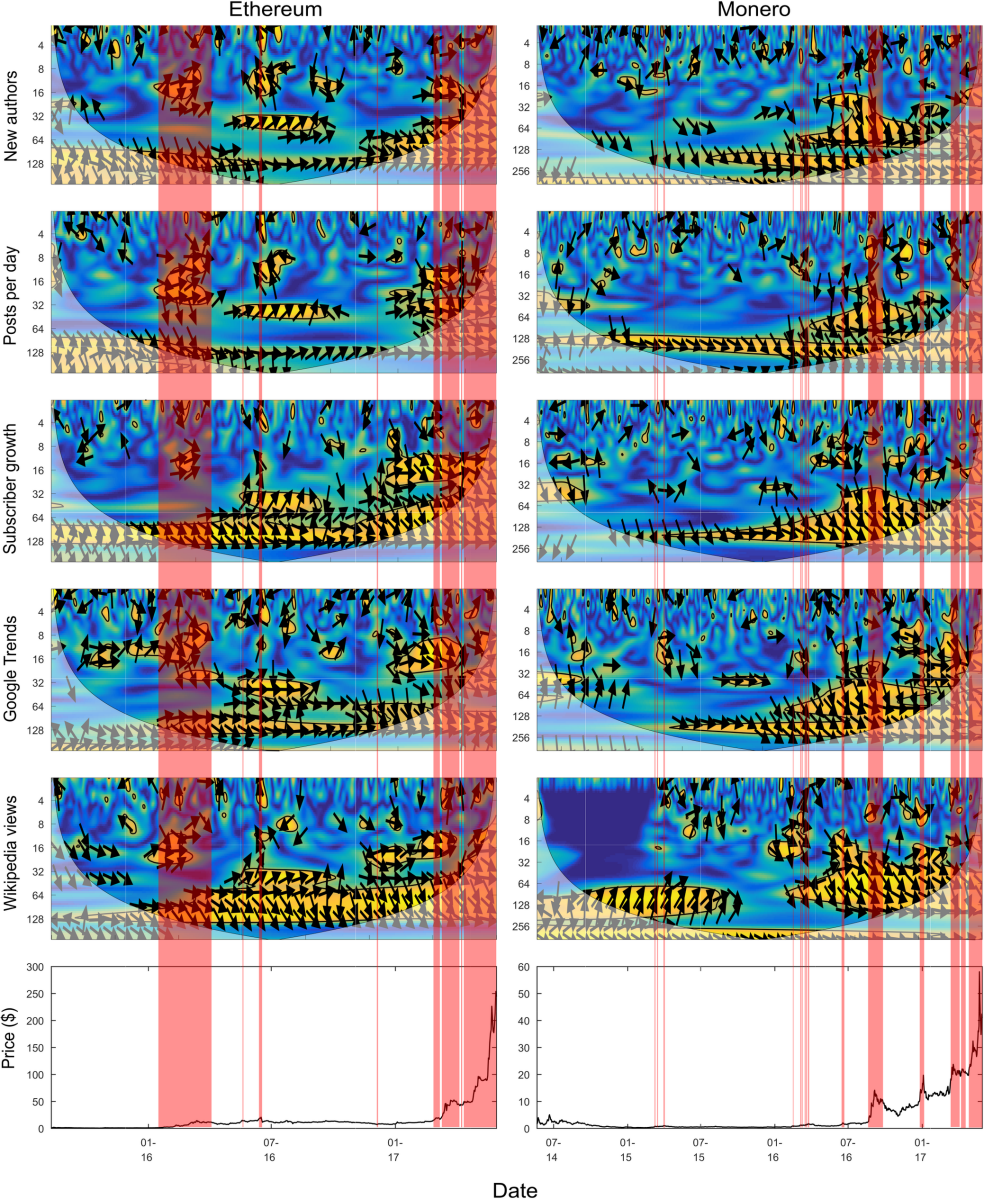
Wavelet coherence scalograms between online factors and price (with GSADF test bubble overlay) for Ethereum and Monero.

**Fig 5:**
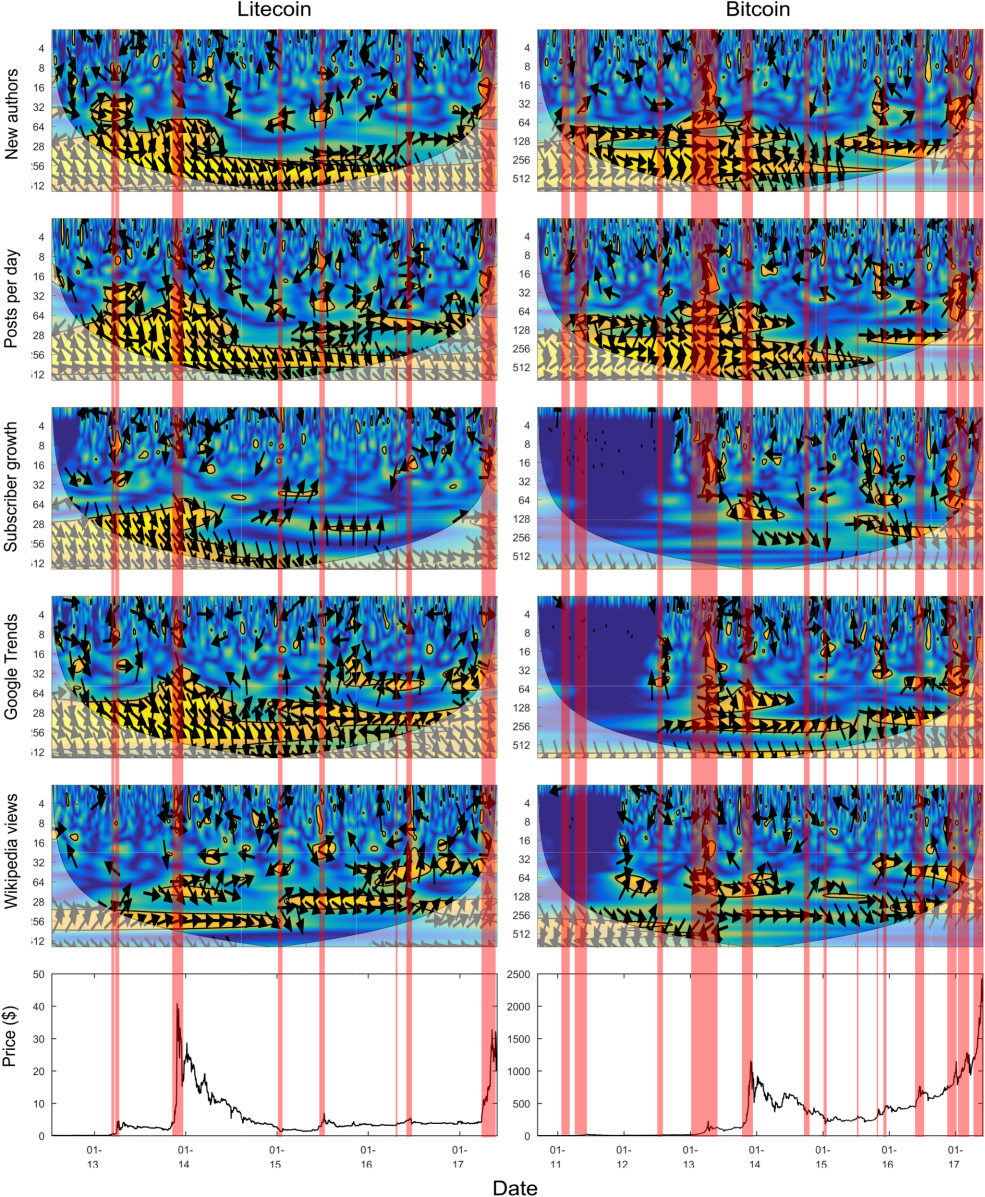
Wavelet coherence scalograms between online factors and price (with GSADF test bubble overlay) for Litecoin and Bitcoin.

Fig 4 and Fig 5 present the wavelet coherence scalograms between the different cryptocurrency and factor combinations. Each column contains scalograms for a different cryptocurrency, each row contains scalograms for a different factor. Looking along a row allows for comparison of any associations between the same factor and different cryptocurrencies. Looking down a column displays how a certain cryptocurrency is associated with different factors. The red shaded areas indicate locations within a cryptocurrency’s price time series that have been identified as in a bubble-like regime, using the GSADF bubble test [24] described previously. It should be noted that the dark blue areas between 2010 and 2012 for Bitcoin subscriber growth, Google Trends, and Wikipedia views are due to a lack of data for these metrics prior to 2012.

For the sake of clarity an explicit definition of short, medium, and long term is required. In this work *short term* refers to the 2–4 and 4–8 day period bands. *Medium term* refers to the 8–16 and 16–32 day bands. *Long term* will be used to refer to the 32–64, 64–128, 128–256 and 256–512 day bands. The short, medium, and long term bands will be considered separately to begin with, and then considered collectively alongside the results of the GSADF bubble test.

#### 3.1.1 Short term relationships

Although short term relationships are erratic and sparse, this is the period band that contains most of the negative—although usually fleeting—relationships (shown by leftward facing arrows). The relationships link online activity increases to price falls (the converse is not observed). It is not surprising that occasionally discussion is associated with price falls, as negative events (e.g. blockchain bugs, and exchange hacks) are newsworthy in the community.

One example is the negative correlation that occurs between Ethereum and its associated factors around June 2016 (left facing arrows at the top and just left of the horizontal middle of the Ethereum scalograms). During this time interval, one of the most well-known applications at the time, the DAO, built on top of the Ethereum environment, was hacked. It can be seen that all factors are negatively correlated in the short term with the price during this time interval. As a result of the uncertainty generated by the hack, price dropped sharply, but activity on social media and interest increased (causing the negative correlation). The negative relationship can be seen during the 2–4 day band for all factors.

In the short term, situations occur where the factors lead the price and where the factors lag the price. However in most cases, the factor lags the price in the short term (seen by upward facing arrows near the top of each scalogram). This is understandable given short term changes appear likely to be the result of particular events, as discussed above. It is likely the market price will reflect the event quicker than social media; social media may experience a longer interval of discussion and activity relating to the original event and resulting price change.

The erratic and spare nature of relationships in the short-term may demonstrate that short term price changes are caused by a confluence of factors, and that online metrics may not be the most interrelated factor with price changes in the short-term. The following examples are given to show what factors can effect cryptocurrency prices in the short-term; both examples are unrelated to the adoption related online metrics considered in this work. Firstly, it is common within cryptocurrency markets for intraday traders to follow technical analysis pattern based trading strategies. Enough traders following these will cause short term price changes based on the indicators they are watching (if enough traders buy believing the price will go up, this will become self-fulfilling). Secondly, as documented later in Section 3.2, there exists isolated periods of short-term coherence between different cryptocurrency prices. Examining cryptocurrency specific online metrics without regard to the general cryptocurrency ecosystem may not provide a complete picture. For example, if a favourable news article occurs for, say, Ethereum, the price of Ethereum may go up, while the price of Bitcoin may go down, as people sell Bitcoins to buy Ethereum. This short-term movement of the Bitcoin price may be unexplainable by Bitcoin related online metrics.

#### 3.1.2 Medium term relationships

Relationships in the medium term are much less erratic than those observed in the short term. Considering all scalograms together, there are distinct patches of strong relationships separated by substantial areas with no relationship present. The relationships are predominately positively correlated, with the clearest exception being the Ethereum DAO hack (June 2016) discussed above, which displays negative medium term correlation for the new authors and posts per day factors (seen in the 8–16 day band just left of the horizontal middle of the Ethereum scalograms). In the medium term, there is no consistency regarding whether it is the factor or the price which leads the observed relationships. Section 3.1.4 below considers the bubble regime overlay and gives an explanation for the temporal emergence of medium term relationships.

#### 3.1.3 Long term relationships

Longer term relationships appear more consistent over time and do not appear directly affected by individual news items. Almost all long term relationships are consistently positive, when they exist; suggesting a positive long term relationship between price and online activity. The lack of consistency of Wikipedia views and consistency of Reddit factors in leading the prices indicate that the Reddit derived factors are better predictive indicators in the long term. Posts per day, new authors, and subscriber growth (all the metrics derived from Reddit) are predominately leading the price in the long term (shown by largely downward oriented arrows). In contrast, Google Trends has more locations where there is no obvious leader and Wikipedia views has more variations than the other factors. There is no consistent leader in the relationships with Bitcoin and Litecoin. For the other two cryptocurrencies considered there are intervals where Wikipedia views significantly lag the Monero price, but in contrast, Wikipedia views lead the Ethereum price throughout the data interval considered.

The long term positive coherence relationship observed between online metrics and price may be the result of another factor which we hypothesise could be technical progress. As a project makes technical progress, it is likely to have a community form around it over time, increasing online activity and also demand, and hence price, of the particular cryptocurrency. An interesting avenue of future work would be to consider the coherence between price and technical progress (via looking at each projects source code repository—these are available as cryptocurrency projects are generally open-source).

#### 3.1.4 Bubble regimes and changing factor relationships

Looking at the bubble regimes (shaded red areas) identified by the GSADF test, it appears there is a strengthening of the medium term—and to some extent long term—coherence relationships within the time intervals identified as being bubble-like regimes; this can be justified intuitively by considering that interest is likely to rise as price rises. This result echoes other work which found that social media factors and price are likely to exhibit positive feedback loops [4], whereby increasing social media usage causes price to increase and vice versa, reinforcing one another. The strengthening of medium term relationships can be seen, to different extents, for all of the factors considered. An example of this is Ethereum between January 2016 and April 2016 (seen in the left most red shaded area of the Ethereum scalograms) where during a prolonged interval identified as a bubble, positive coherence forms between all factors (most prominently in posts per day) and the price.

Long term relationships also strengthen, to some extent, around areas indicated as bubbles. The previously observed long term relationship between Google Trends and Bitcoin price [8] can also be seen here, between late 2012 and 2014 (period band 64–256). With the benefit of extra data it can be observed that the relationship disappears around 2014 (for lower period bands) and 2015 (for higher period bands), before the relationships start occurring more consistently in 2016 and 2017 (a region with a number of bubbles identified). The previously observed relationship between Wikipedia views and Bitcoin observed in 2013 (64–128 band), disappears before again returning in mid-2016 and 2017.

Three of the factors considered here (new authors, posts per day, and subscriber growth) have been used in previous work by us [7] to predict bubbles in the price series. The two most prominent areas of bubble-like behavior identified in this previous work were 1) in the Ethereum price between January and April 2016, and 2) in the Monero price in August/September 2016. Both of these areas are also identified as bubbles in the current work using the GSADF test, and are appropriately shaded red in Fig 4. The identification here of both regions as being in the bubble region adds credence to their identification as bubble regimes in [7], which did not consider the price series, only social media usage. It is insightful to consider each of these bubbles separately. For the Ethereum early 2016 bubble, it can be seen that medium term relationships form during this interval for all three Reddit factors. For the Monero August/September 2016 bubble, it can be seen that medium to long term relationships strengthen between the three factors and the price. Furthermore the factors appear to be consistently leading the price series, making them good predictors. The analysis here establishes in a model independent fashion that tangible relationships are present in the bubble regions identified by the previous work.

**Fig 6:**
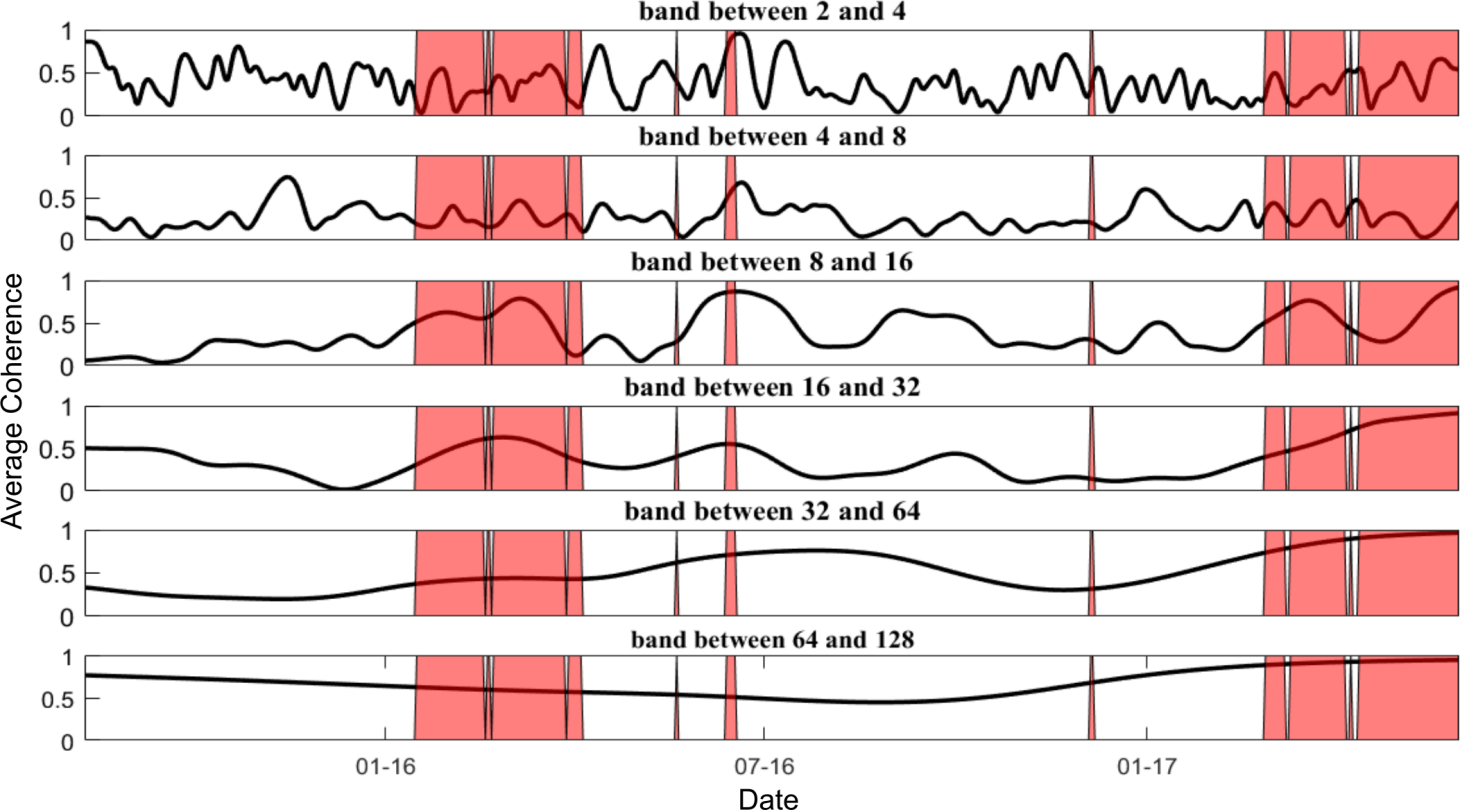
Wavelet coherence between Ethereum new authors and price decomposed for different period bands (with GSADF test bubble overlay).

Interpretation of visual scalograms is subjective so it is desirable to find a more quantifiable way to validate the strengthening of coherence in bubble regimes. Fig 6 shows the wavelet coherence over time for the different period bands, in the case of the “new authors” factor for Ethereum. Coherence values, plotted on the vertical axis, vary between zero and one. Time is plotted on the horizontal axis. The areas of the price time series that are recorded as bubble-like regimes using the GSADF test are shaded red.

It can be seen from Fig 6 that coherence in the short run is erratic throughout the time interval analysed, and that there is little appreciable difference between the bubble and non-bubble regimes. However in the medium term (8–16 and 16–32 days), coherence generally peaks around areas where bubbles have been identified in the price series. The longer term relationship, though, is less dependent on whether the price is in a bubble phase.

**Fig 7:**
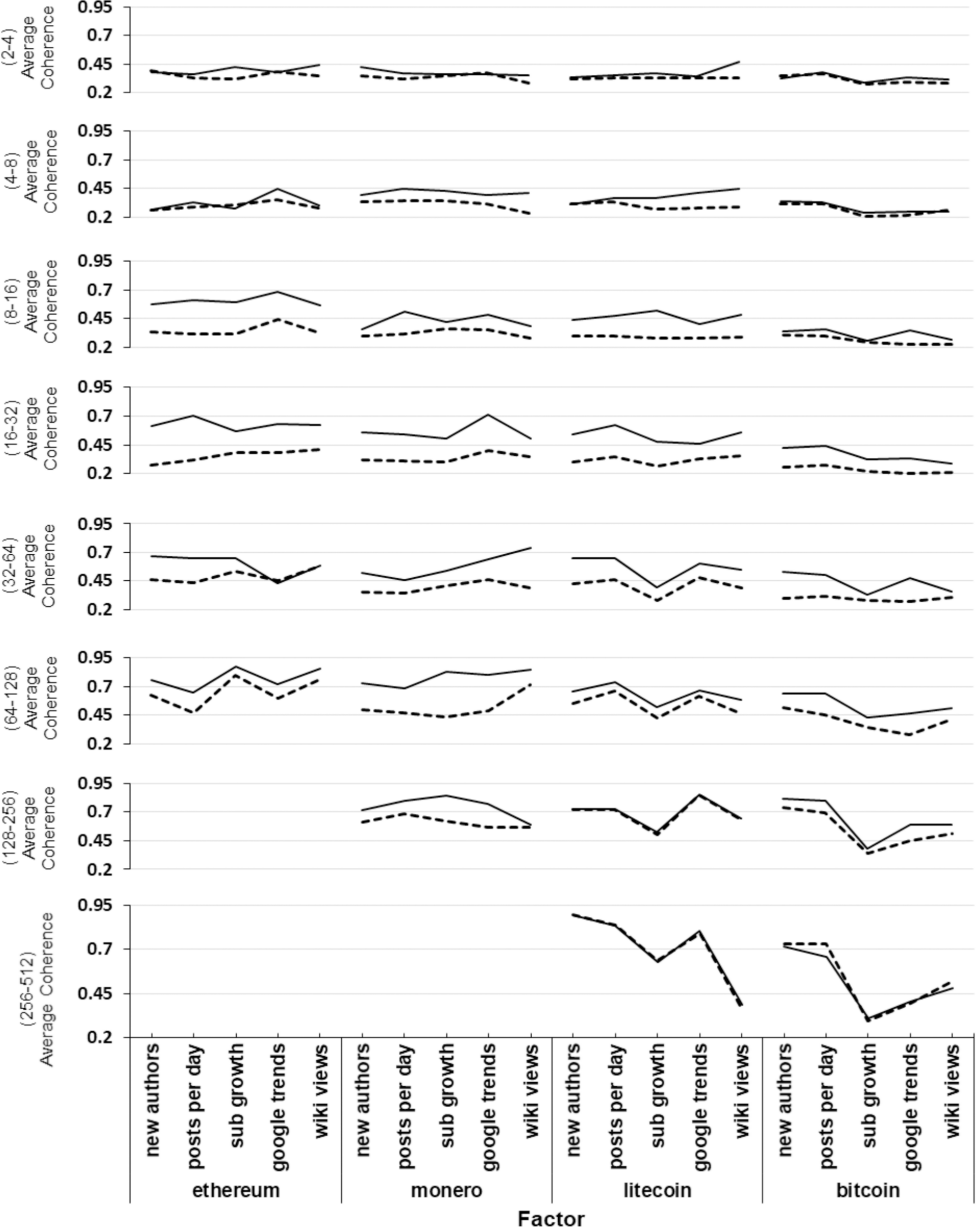
Visualisation of the average wavelet coherence values for bubble (solid) and non-bubble (dashed) regimes decomposed by period band.

Although analysis of a single factor and cryptocurrency combination, as above, is of interest, more general findings across multiple cryptocurrency/factor combinations can also be pursued. Fig 7 shows, for each cryptocurrency and factor combination, the mean coherence values during the bubble and non-bubble regimes. Each horizontal subplot shows a different coherence period band, from the lowest period band (2–4 days) at the top to the highest period band (256–512 days) at the bottom. As the duration of data for each cryptocurrency varies, certain ranges are left blank when that cryptocurrency does not have enough data to produce values for such bands.

From Fig 7 it can be seen that, for all cryptocurrency/factor combinations, there is very little difference in coherence values between the bubble and non-bubble regimes in the 2–4 day band. In the 4–8 band, some differences are observed, but without consistency (there are occurrences of bubble regime coherence values being below the non-bubble regime values). In the 8–16 and 16–32 day period bands, large differences can be seen in the coherence values between the bubble and non-bubble regime (for all factors), with the bubble regime coherence being consistently above the non-bubble regime coherence. Ethereum exhibits the largest medium term (8–16 and 16–32) differences in coherence values between its factors for bubble and non-bubble regimes. The differences observed start to reduce as the period bands get larger (with the exception of Monero which exhibits longer term differences). Almost all impact of the bubble regime has disappeared by the 256–512 data band (for those cryptocurrencies with enough data to generate results), where very similar values are seen for the bubble and non-bubble regimes.

It can in addition be observed from Fig 7 that as the period band considered increases, the overall (bubble and non-bubble) coherence values generally get stronger, suggesting online factors have a medium to long term link with price.

Bitcoin’s coherence values appear noticeably less affected by bubble and non-bubble regimes, especially over short and medium terms (2–4, 4–8, 8–16 and 16–32). The non-bubble coherence values are similar to those of the other cryptocurrencies, but the bubble regime values do not reach a similar magnitude to the other cryptocurrencies.

It appears that there are a number of potential explanations for this. Bitcoin has always been the most well-known cryptocurrency, and so online activity that appears related to it may actually be about cryptocurrencies in general (rather than specific to Bitcoin), resulting in less of a relationship between this perceived activity and the Bitcoin price. Furthermore, the Bitcoin subreddit considered in this work (/r/Bitcoin) is commonly used as a platform for the community to debate a variety of contentious scaling solutions that would enable the Bitcoin network to process more transactions concurrently. The amount of activity debating scalability would be unlikely to change dramatically in relation to price changes; potentially providing further reason for the lack of strengthening of Bitcoin’s coherence with Reddit based factors in bubble-like regimes of the price series.

To validate whether the coherence values observed in the bubble and non-bubble regimes are statistically different, a two-sample one-tailed t-test is conducted (for each cryptocurrency / metric pair). A one-tailed test is chosen as it is only of interest whether the coherence values in the bubble regime are statistically larger than in the non-bubble regime. The null hypothesis is that there is no statistically significant difference between the coherence values in bubble and non-bubble regimes and the alternative hypothesis is that the coherence values in the bubble regime are statistically larger than the non-bubble regime. The t-test p-values are listed in Table 2. The cells with a p-value smaller than 0.01 are highlighted grey; in such cases the null hypothesis can be rejected in favour of the alternative hypothesis.

**Table 2:** T-test p-values (for each period band of each cryptocurrency / metric pair).

Cryptocurrency	Metric	Period band							
		2–4	4–8	8–16	16–32	32–64	64–128	128–256	256–512
Ethereum	New authors	0.200	0.323	0.000	0.000	0.000	0.000		
	Posts per day	0.017	0.001	0.000	0.000	0.000	0.000		
	Subscriber growth	0.000	0.005	0.000	0.000	0.000	0.000		
	Google trends	0.219	0.000	0.000	0.000	0.068	0.000		
	Wikipedia views	0.000	0.023	0.000	0.000	0.420	0.000		
Monero	New authors	0.000	0.000	0.000	0.000	0.000	0.000	0.000	
	Posts per day	0.000	0.000	0.000	0.000	0.000	0.000	0.000	
	Subscriber growth	0.170	0.000	0.000	0.000	0.000	0.000	0.000	
	Google trends	0.282	0.000	0.000	0.000	0.000	0.000	0.000	
	Wikipedia views	0.000	0.000	0.000	0.000	0.000	0.000	0.103	
Bitcoin	New authors	0.005	0.001	0.000	0.000	0.000	0.000	0.000	0.070
	Posts per day	0.146	0.029	0.000	0.000	0.000	0.000	0.000	0.000
	Subscriber growth	0.059	0.001	0.162	0.000	0.000	0.000	0.002	0.000
	Google trends	0.000	0.000	0.000	0.000	0.000	0.000	0.000	0.093
	Wikipedia views	0.001	0.082	0.000	0.000	0.000	0.000	0.000	0.000
Litecoin	New authors	0.256	0.454	0.000	0.000	0.000	0.000	0.209	0.395
	Posts per day	0.031	0.016	0.000	0.000	0.000	0.000	0.380	0.415
	Subscriber growth	0.002	0.000	0.000	0.000	0.000	0.000	0.175	0.364
	Google trends	0.051	0.000	0.000	0.000	0.000	0.009	0.143	0.023
	Wikipedia views	0.000	0.000	0.000	0.000	0.000	0.000	0.029	0.027

It can be observed that in the short term (2–4 and 4–8 day period band) there is no consistency in results; in some cases the null hypothesis can be rejected and in some cases it cannot. In the medium term there is more consistency in rejection of the null hypothesis in favour of bubble regime coherence values significantly exceeding the non-bubble regime values. In the long term, the proportion of instances exhibiting statistical significance reduces, with the majority of cases in the 256–512 band not being a rejection of the null hypothesis. This reduction of statistically significant differences when considering longer term periods further emphasises the point that it is the medium term in which coherences tend to strengthen during bubble regimes.

### 3.2 Coherence between different cryptocurrencies

An interesting avenue to explore is the wavelet coherence between different cryptocurrencies, allowing any relationships between different cryptocurrencies to be detected and documented. Relationships between different cryptocurrencies would be of interest for those searching for diversification within cryptocurrency markets, especially to those managing a portfolio of cryptocurrencies.

**Fig 8:**
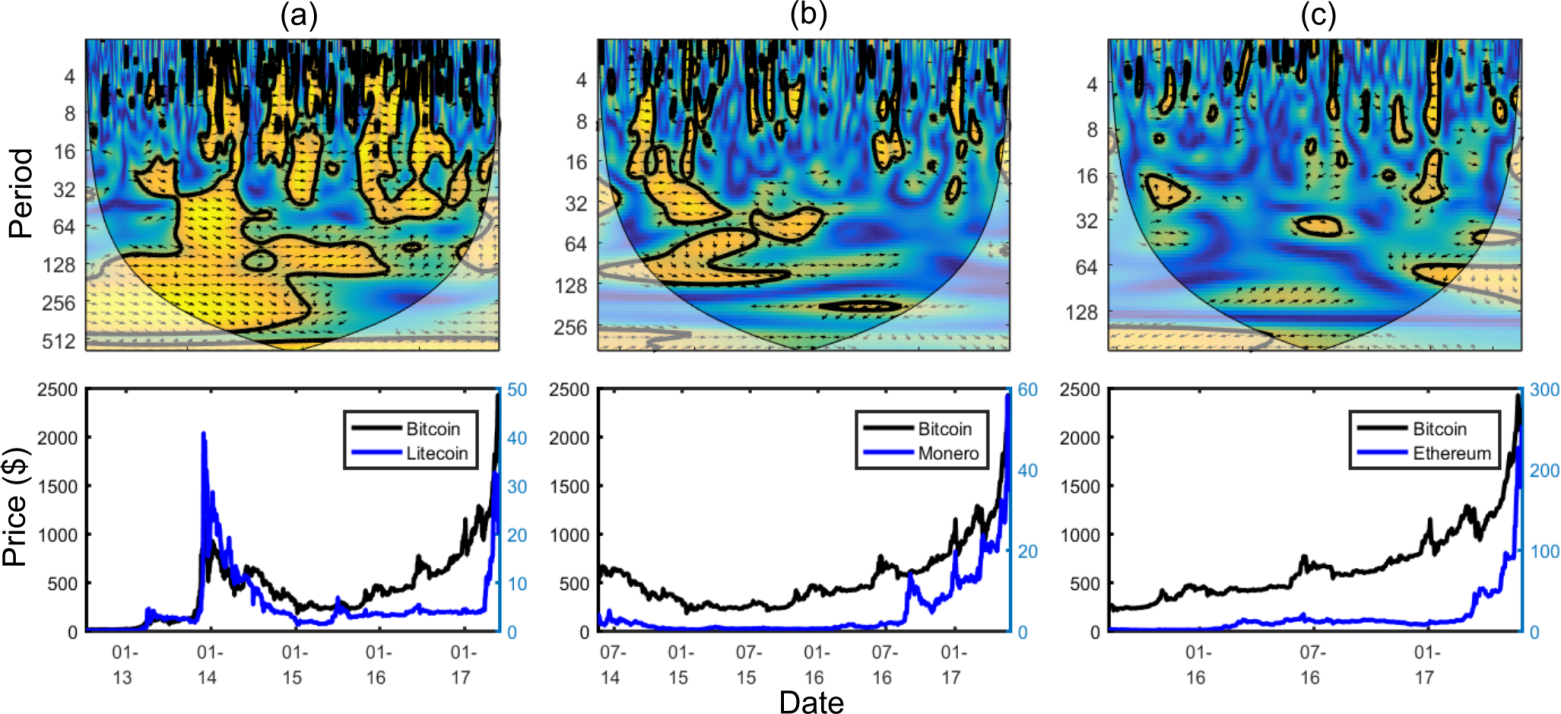
Wavelet coherence plots between (a) Bitcoin and Litecoin prices; (b) Bitcoin and Monero prices; (c) Bitcoin and Ethereum.

Fig 8(A) shows many significant positive correlations between Bitcoin and Litecoin. This is an expected relationship given Litecoin is technically very similar to Bitcoin (Litecoin is essentially Bitcoin with faster block confirmations). Overall, there is no clear leader in the relationship. However during the interval of the late 2013 price bubble (where Bitcoin and Litecoin reached around $1000 and $40 respectively) it can be seen that Bitcoin is leading Litecoin (slightly downward facing arrows across all periods).

The longer term relationship varies over time. After exhibiting strong positive correlation in 2013 (where prices rose), and 2014 (where prices fell for a sustained interval), the longer term relationship between Bitcoin and Litecoin starts to break down around the middle of 2015. It can be seen in the accompanying price plot that at this point the Bitcoin price starts to gradually recover, whereas the Litecoin price does not.

Over the short and medium term there are frequent intervals of positive correlation between Bitcoin and Litecoin. There is a limited interval, around March 2017, where no significant relationship exists in the short term (the top right of Fig 8(A)), where a positive relationship had previously existed. This lack of positive relationship suggests the price movements decoupled. Two reasons could contribute towards this decoupling. In early March, the SEC gave its long awaited decision on a Bitcoin ETF (investment vehicle), but it appears this had little impact on Litecoin; Litecoin was potentially even used as a hedge against the resulting Bitcoin price changes. In late March, percentage support for a Litecoin technical enhancement (SegWit) increased beyond the threshold percentage required for adoption around the same time as significant increases in the Litecoin price. The adoption of this change would temporarily reduce the similarity between Bitcoin’s and Litecoin’s technology (Bitcoin has since also adopted SegWit).

Fig 8(B) and 8(C) show less consistent relationships with Bitcoin. Fig 8(B) shows that Monero nearer its inception was significantly impacted by Bitcoin price changes (positive correlation with Bitcoin leading the price changes (seen towards the left of Fig 8(B)), with co-movement over the short, medium, and long terms. In 2016, Monero had a number of positive developments which may have led to its price behaviour decoupling from Bitcoin’s. For example, on August 22^nd^, AlphaBay Market, a dark-net market, announced they would start accepting Monero-based transactions. Integration announcements from other dark-net markets also occurred around this time prompting mainstream media coverage. Furthermore, as Monero grows, a lack of long-term co-movement is understandable due to very different objectives to Bitcoin (unlike Litecoin and Bitcoin which have very similar objectives); Monero focusses primarily on privacy of those transacting whereas Bitcoin does not.

There is a lack of longer term relationship between the Bitcoin and Ethereum price (Fig 8(C)). Although there are limited areas of co-movement, there is no clear pattern. However the short term exhibits brief intervals of co-movement. It is likely that events that affect the cryptocurrency environment as a whole will have similar (short-term) effects on all cryptocurrencies. One example in early January 2017 can be examined to demonstrate this. Following weeks of increasing Bitcoin prices (and high volatility) while trading around its all-time high, on January 6^th^ 2017, the People’s Bank of China (PBOC) issued a statement expressing their concern regarding Bitcoin’s recent price volatility, and reminding cryptocurrency exchanges that they must operate within the laws and regulations of China. This caused cryptocurrency markets to speculate a tightening of regulations was imminent and was especially significant as, at the time, Chinese trading was reported to be around 95% of global Bitcoin trading volume. The price of many cryptocurrencies decreased during this period. This example highlights how individual events have a similar impact on a number of cryptocurrencies (and hence, short-term positive coherence). The resulting positive coherence can be seen on a short-term horizon for both Monero (8 (b)) and Ethereum (8(c)) around early 2017 (the strips of yellow, touching the top most border towards the top right of the scalograms).

Overall, it appears from these results that cryptocurrencies may experience short term intervals of co-movement, caused by sector wide news or cross market contagion, though correlation is likely to change dependent on the nature of the causal event and market environment. In the medium and longer term Bitcoin and Litecoin are strongly related; it is believed this is due to their similarity.

## 4 Conclusions

The use of wavelets in this work has demonstrated how factor relationships are prone to strengthen and weaken their correlation with price as a cryptocurrency goes through different market regimes (specifically, in this case, bubbles). The main finding is that medium term relationships with online factors strengthen during cryptocurrency price bubbles. Using the results of the GSADF test overlaid on the scalograms together with further analysis provides some explanation of why the medium term relationships strengthen when they do. These findings will be of use to anyone exploring factor dependence of cryptocurrencies in either an academic or industry setting. The strengthening of relationships during bubbles also demonstrates how cryptocurrencies may currently be used as speculative assets (among other use cases), as price increases are usually associated with increases in online activity (which can be assumed to represent interest).

The strengthening of coherence in bubble regimes is much less prominent in the short and long term. In the short term, the effect of bubbles may be hidden by the effects of daily news items and intraday trading activity. It is also seen that in the short term the relationship between online factors and cryptocurrency prices are erratic and generally weak; there is little consistency as to whether the price or factors are leading, though slightly more negative relationships exist in this period band. The erratic relationships over the short term suggest online factors may not be best predictor in the shorter term.

Online factors exhibit stronger relationships in the long term, and such relationships were found to be consistently positive. The long term positive relationships suggest long term price trends are linked with online activity. This is an intuitive result, given that successful cryptocurrencies are likely to have active communities; as the community grows, so does belief in the cryptocurrency, and vice versa.

Turning to the relationships between different cryptocurrencies, significant coherence is observed in the medium and long term between Bitcoin and Litecoin, which it is believed is due to their similarity. It is seen that short term correlations between the cryptocurrencies considered here are dependent again on news items and market wide events.

In summary it is hoped that the findings presented here will motivate further work in the area, especially relating to bubble dynamics within cryptocurrency markets, and, separately, as to how factor relationships change over time. Furthermore, the short term relationships inferred between cryptocurrencies and news items could justify investigation into the mechanisms by which events such as news items affect cryptocurrency markets (possibly in a similar manner as already studied for other asset classes [26]), and could result in a portfolio balancing trading strategy automatically adjusting to market news. It should also be noted that three of the metrics used here—posts per day, subscriber growth and new authors—are recorded from the social media platform Reddit. The work here, along with [7], has demonstrated the possibility of using Reddit activity to predict cryptocurrency prices. Further research into the relationship between Reddit and cryptocurrencies could involve sentiment analysis, a comparison between the predictive power of Reddit compared to Twitter in cryptocurrency markets, and variants of models based on user reputation.
